# Hyponatremia Is Associated With Urinary Retention: The Vasopressin-Dependent and -Independent Pathways

**DOI:** 10.7759/cureus.88933

**Published:** 2025-07-28

**Authors:** Ririna Demizu, Tsuneaki Kenzaka, Ryo Nishio, Hogara Nishisaki

**Affiliations:** 1 Department of Internal Medicine, Hyogo Prefectural Tamba Medical Center, Tamba, JPN; 2 Division of Community Medicine and Career Development, Kobe University Graduate School of Medicine, Kobe, JPN

**Keywords:** case report, hyponatremia, secretion of inappropriate antidiuretic hormone, urinary retention, vasopressin

## Abstract

Hyponatremia has numerous causes, and the vasopressin-independent and vasopressin-responsive pathways have been associated with the condition. Herein, we aimed to present a case of hyponatremia induced by urinary retention, a rare cause of hyponatremia. A woman in her 80s presented to the emergency department with malaise and difficulty in movement. Plasma sodium levels of 118 mEq/L, urinary retention, and an *Escherichia coli* urinary tract infection were detected. Her condition and plasma sodium levels resolved through urinary catheterization, sodium administration, and antibiotic treatment. Evaluating serum vasopressin levels aids in differentiating between urinary retention and hyponatremia, and urinary retention treatment is crucial regardless of the etiology.

## Introduction

Hyponatremia is defined as a serum sodium concentration of <135 mEq/L. Hyponatremia is a common fluid and electrolyte imbalance encountered in clinical practice. The usual causes of hyponatremia include the syndrome of inappropriate antidiuretic hormone secretion (SIADH), congestive heart failure, water overload, the postoperative state, and use of certain drugs [1].

Urinary retention is a newly identified trigger for hyponatremia [2,3]. Hyponatremia and urinary retention are common in older individuals; however, this is largely unexplored. Therefore, the relationship between the two conditions remains poorly understood. In this report, we aim to present a case of hyponatremia induced by urinary retention.

The inappropriate secretion of antidiuretic hormone (ADH) in response to bladder distension is a proposed mechanism [2]. This hypothesis is supported by case reports showing that the relief of urinary retention results in a rapid improvement of hyponatremia [4,5].

## Case presentation

A woman in her 80s with a slight decline in cognitive function for several years was found on the kitchen floor and transferred to the emergency department (ED). The patient presented with malaise and difficulty in movement. According to her son, the patient had been in her usual state of health the previous day. The blood tests conducted one month earlier showed no unusual findings in the blood parameters, including the serum sodium levels. The medical history included hyperlipidemia, gastroesophageal reflux disease, lower back pain, knee osteoarthritis, and hypertension. The patient was not on diuretics. Her evaluation at the ED indicated a temporal temperature of 37.6 °C, blood pressure of 163/98 mmHg, a pulse of 93 bpm, and an oxygen saturation at ambient air of 98%. The Glasgow Coma Scale was E3V5M6. On physical examination, the patient appeared euvolemic, with no signs of volume overload or dehydration. A slight inferior vena cava collapse, distended bladder, bilateral hydronephrosis, and dilated ureters were detected on abdominal ultrasonography.

Blood test results showed a white blood cell count of 13,890/µL, neutrophil ratio of 90.7%, C-reactive protein level of 2.12 mg/dL, blood urea nitrogen level of 21.9 mg/dL, creatinine level of 0.65 mg/dL, sodium level of 118 mEq/L, potassium level of 4.6 mEq/L, and chloride level of 102 mEq/L. Therefore, hyponatremia (118 mEq/L) and an elevated inflammatory response were detected during the initial evaluation at the ED (Table 1).

**Table 1:** Laboratory data at the emergency department

Parameter	Recorded value	Standard value
White blood cell count	13,890/µL	4500–7500/µL
Neutrophils	90.7%	42–74%
Hemoglobin	11.9 g/dL	11.3–15.2 g/dL
Platelet count	18.8 × 10^4^/µL	13–35 × 10^4^/µL
C-reactive protein	2.12 mg/L	≤0.60 mg/dL
Total protein	7.3 g/dL	6.9–8.4 g/dL
Albumin	4.4 g/dL	3.9–5.1 g/dL
Total bilirubin	1.2 mg/dL	0.2–1.2 mg/dL
Aspartate aminotransferase	40 U/L	11–30 U/L
Alanine aminotransferase	29 U/L	4–30 U/L
Lactase dehydrogenase	440 U/L	109–216 U/L
Creatine kinase	130 U/L	40–150 U/L
Blood urea nitrogen	21.9 mg/dL	8–20 mg/dL
Creatinine	0.65 mg/dL	0.63–1.03 mg/dL
Sodium	118 mEq/L	136–148 mEq/L
Potassium	4.6 mEq/L	3.6–5.0 mEq/L
Chloride	86 mEq/L	98-108 mEq/L
Glucose	157 mg/dL	70–109 mg/dL
Hemoglobin A1c	5.6%	5.6–5.9%

Additional laboratory test findings revealed a plasma osmolarity of 247 mOsm/L, urine osmolarity of 358 mOsm/L, and urine sodium concentration of 51 mEq/L. Thyroid-stimulating hormone, free thyroxine, and cortisol levels were 2.42 μIU/mL, 1.03 ng/dL, and 18.7 μg/dL, respectively (Table 2).

**Table 2:** Additional laboratory data

Parameter	Recorded value	Standard value
Thyroid-stimulating hormone	2.42 μIU/mL	0.61-4.23 μIU/mL
Free thyroxine	1.03 ng/dL	0.70-1.48 ng/dL
Cortisol	18.7 μg/dL	
Uremic acid	3.1 mg/dL	2.6-5.5 mg/dL
Renin	0.8 ng/mL/h	
Aldosterone	4.9 pg/mL	4.0-82.1 pg/mL

**Figure 1:**
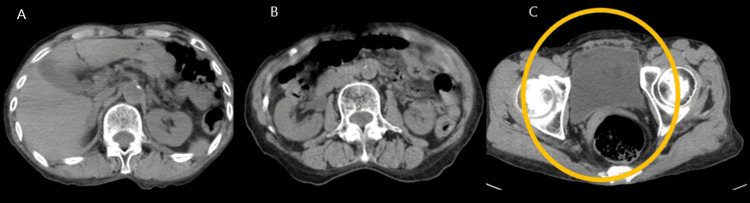
Noncontrast CT of the abdomen. (A) CT image showing no signs of adrenal abnormality. (B) Ureteral dilation is observed on the CT image; however, there are no visible ureteral stones. (C) CT image showing bladder distention with urinary retention. The yellow circle indicates urine retention in the bladder. CT: computed tomography

Urinalysis revealed bacteriuria. *Escherichia coli* (*E. coli*) was detected in the urine culture on the following day, while no bacterial growth was observed on blood cultures. Head computed tomography (CT) and chest radiography revealed no apparent abnormalities. Abdominal and pelvic CT revealed no adrenal abnormalities or malignant pathologies, except bladder distension (Figure 1); however, the image was taken after draining 700 mL of urine with a straight catheter at the ED. Ureteral dilation was observed on CT; however, there were no visible ureteral stones (Figure 1).

Differential diagnoses of SIADH and mineralocorticoid-responsive hyponatremia of the elderly were made based on the euvolemic (or slightly hypovolemic, supposedly owing to urinary tract infection) state and elevation in urine sodium levels. Owing to the low plasma levels of uremic acid, renin, and aldosterone, SIADH was primarily suspected (Table 2).

**Figure 2:**
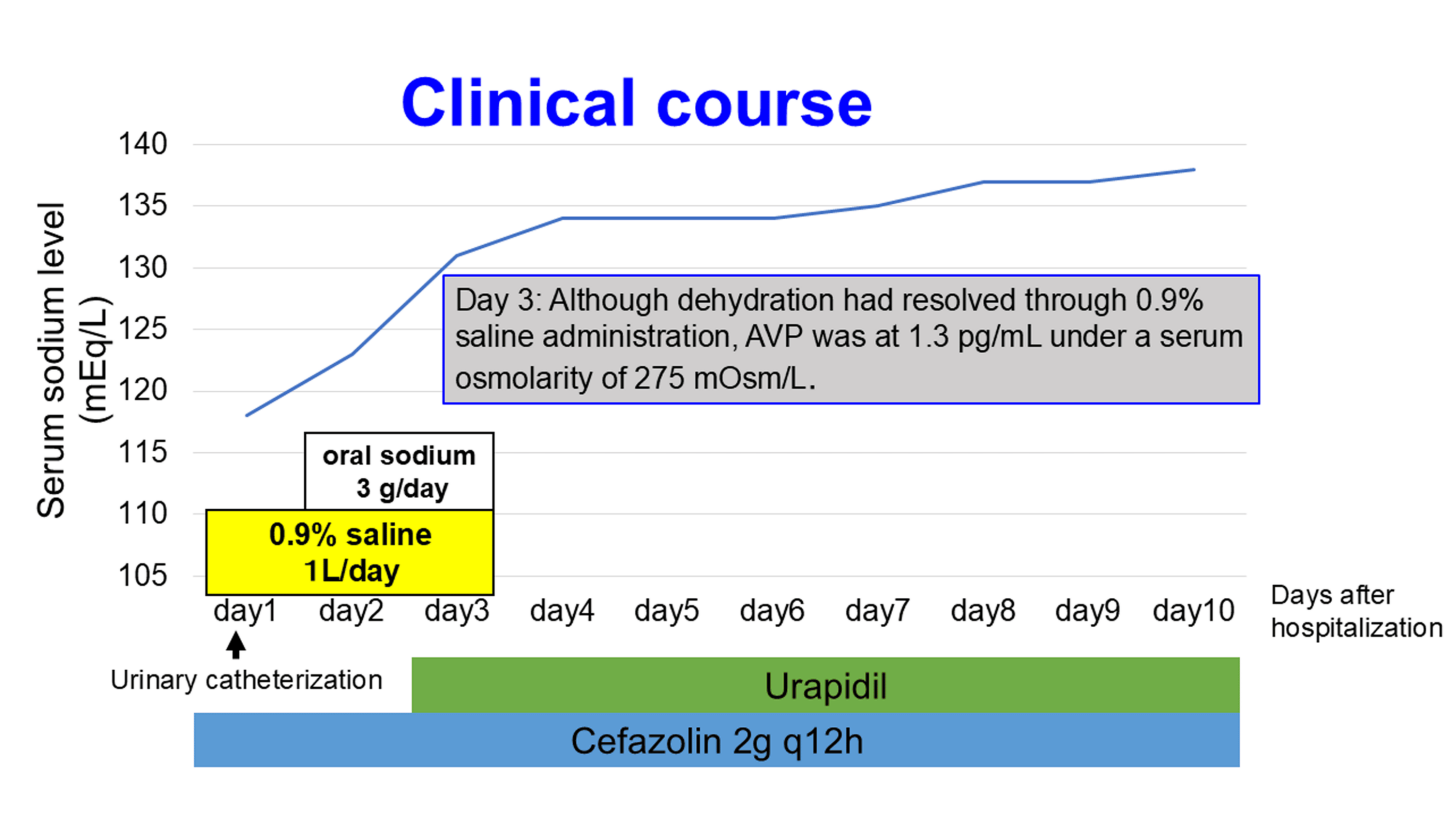
Clinical course of serum sodium levels. AVP: arginine vasopressin

Owing to severe hyponatremia, 1 L of intravenous 0.9% saline and oral sodium were administered to the patient at the ED. The plasma sodium levels increased to 124 mEq/L after 16 hours. In addition, 2 g of cefazolin was administered intravenously twice daily to treat E. coli bacteriuria for 14 days. Urethral catheterization was not performed after admission because the patient had spontaneous urination. However, there was approximately 300 mL of residual urine after micturition, prompting urological consultations. The patient had an underactive bladder, and urapidil treatment was initiated by a urologist. On the third day of hospitalization, following dehydration correction, plasma osmolarity and arginine vasopressin levels were assessed to determine the cause of hyponatremia. Despite low serum osmolarity (275 mOsm/L) and serum sodium levels of 130 mEq/L, arginine vasopressin was detected (1.3 pg/mL). If serum osmolality is below 280 mOsm/L, serum arginine vasopressin is usually below the detection limit. The initial sodium correction was ceased on this day. By the fourth day of hospitalization, the urinary retention had completely resolved (with no residual urine upon ultrasonography), and the plasma sodium levels were normal without any sodium administration since the fourth day. Therefore, we concluded that the patient had SIADH. Figure 2 shows the treatment and serum sodium levels during hospitalization.

The patient was discharged on Day 15. No causes of SIADH other than urinary retention were found. Urinary retention improved with urological medications, and no hyponatremia was noted during the six-month follow-up.

## Discussion

Herein, we present a case of hyponatremia associated with urinary retention. Two underlying etiologies exist, namely the vasopressin-independent [4] and -dependent pathways [2].

Upon urinary retention owing to post-renal failure, the glomerular filtration rate decreases, leading to insufficient water diuresis. In addition, fluid turbulence in the inner medullary collecting duct of the distal nephron aids vasopressin-free reabsorption of free water, leading to vasopressin-independent hyponatremia [4]. With the administration of 0.9% saline and urinary retention treatment by insertion of urinary catheter, the sodium levels of our patient increased sharply from 118 to 124 mEq/L in 16 hours.

SIADH has a vasopressin-responsive etiology. Pain and vagal nerve stimulation triggered by elevated intravesical pressure cause excessive vasopressin secretion [2].

Despite hyponatremia, low serum osmolality, and a urine osmolality of >100 mOsmol/kg, arginine vasopressin is detected in patients with SIADH. When the urine sodium concentration is >40 mEq/L, the serum potassium levels are normal, whereas the serum uric acid levels are low. Hence, excluding adrenal insufficiency and severe hypothyroidism is essential [6,7]. Our case met these criteria, leading to a SIADH diagnosis.

## Conclusions

We presented a case of urinary retention-induced hyponatremia, which is attributed to vasopressin-independent and vasopressin-responsive pathways. Evaluating serum vasopressin levels facilitates the differentiation of these conditions. Hyponatremia could be vasopressin independent; however, the diagnosis implicated SIADH in this case. A significant limitation is that these are reported only in case reports; thus, studies with a larger population are warranted. Furthermore, urinary retention treatment remains crucial regardless of the etiology.
